# Outpatient antibiotic stewardship during the COVID-19 era: analysis of prescribing trends and guideline compliance

**DOI:** 10.1017/ash.2025.10081

**Published:** 2025-08-04

**Authors:** Minji Sohn, Benjamin Pontefract, Kushal Dahal, Michael Klepser

**Affiliations:** 1 College of Pharmacy, Ferris State University, Big Rapids, Michigan, USA.; 2 Collaborative to Advance Pharmacy Enterprises (CAPE), Ferris State University, Grand Rapids, Michigan, USA.

## Abstract

**Objective:**

To analyze antibiotic prescribing trends and guideline concordance in outpatient settings using electronic health records (EHRs).

**Design:**

This quality improvement study utilized data from the Collaboration to Harmonize Antimicrobial Registry Measures (CHARM) database, which integrates antibiotic prescribing data extracted from the EHRs of various outpatient facilities.

**Setting:**

The study was conducted across 352 outpatient facilities in the United States.

**Participants:**

The study included oral antibiotic prescribing data from outpatient encounters from January 2021 to June 2023, encompassing 823,938 prescriptions.

**Methods:**

The primary outcomes were the rate of antibiotic prescribing per 1 000 prescription-related outpatient visits and identifying frequently prescribed antibiotics in adults and children. Secondary outcomes were the prescribing patterns for selected diagnoses and the concordance of these prescriptions with published guidelines.

**Results:**

The study estimated approximately a 20% increase in antibiotic prescribing per year, with an overall rate of 121.26 prescriptions per 1 000 prescription-related outpatient visits (95% confidence interval 121.01–121.50). Amoxicillin-clavulanate, amoxicillin, doxycycline, and cephalexin were most frequently prescribed. Sinusitis and otitis media were the most common reasons for prescribing antibiotics among adults and children, respectively. Less than 60% of sinusitis-related prescriptions were antibiotic concordant. Duration concordance rates were less than 70% for sinusitis, urinary tract infections, cellulitis, and Group A Streptococci. 51% of ciprofloxacin prescriptions were for patients aged 60 or older.

**Conclusions:**

The findings stress the need for strengthened antimicrobial stewardship in outpatient settings. The increasing rate of antibiotic prescriptions and discrepancies in guideline concordance reiterate the importance of ongoing monitoring and targeted interventions.

## Introduction

Antibiotic resistance is a pressing public health treat, with at least 2.8 million antibiotic-resistant infections and over 35,000 deaths annually in the United States.^1^ Misuse and overuse of antibiotics contributed to rising resistance, increased healthcare costs, and unnecessary patient harm.^2–4^ In 2022, 236.4 million antibiotic prescriptions were distributed in outpatient settings, many of which were unnecessary or inappropriate.^5^ To combat this issue, antimicrobial stewardship programs (ASPs) are essential in promoting responsible antibiotic use and curbing resistance.

Since 2020, the Joint Commission has required outpatient ASP performance measures, prompting many facilities to integrate stewardship efforts.^6^ Electronic health records (EHRs) offer valuable insights into prescribing patterns and guideline adherence, facilitating data-driven interventions to optimize antibiotic use.^7–10^

This study analyzes antibiotic prescribing trends and guideline concordance using EHR data from outpatient facilities. We estimate quarterly prescribing rates and identify commonly prescribed antibiotics in adults and children. We evaluate prescribing patterns for selected diagnoses and the concordance of these prescriptions with published guidelines. By examining this trend, we aim to identify potential gaps in clinical practice and areas for intervention to enhance the judicious use of antibiotics.

## Methods

This quality improvement study used the Collaboration to Harmonize Antimicrobial Registry Measures (CHARM) registry database. The CHARM project is a collaborative initiative to standardize antibiotic use measures among healthcare clinics for benchmarking. Through the partnership between an academic institution and healthcare organizations, CHARM investigators at the academic institution receive antibiotic-prescribing data extracted from outpatient clinics’ EHRs and composite it into a standardized database registry. Data-providing facilities receive an interactive dashboard tracking the antibiotic prescribing trend in their facilities and other facilities for comparison.

We compiled the study data set from the CHARM database to include oral antibiotic prescribing data of 352 outpatient facilities in the Midwest region between January 2021 and June 2023. The type of facilities included primary care, urgent care, outpatient specialty, and subspecialty. The data set included the following variables: the date of antibiotic prescribing encounter, the generic name of an antibiotic prescribed, duration of therapy, International Classification of Diseases Tenth Revision (ICD-10), and patient age. In addition to this antibiotic prescribing data, data on the aggregate count of encounters generating one or more prescriptions of any drug in outpatient settings were available. This aggregate data were available for each quarter-year, enabling the estimation of the quarterly antibiotic prescribing rate per 1 000 prescription-related outpatient encounters. The aggregate data was nonspecific to the patient’s age.

We categorized ICD-10 codes into diagnosis groups. Two clinicians with expertise in infectious diseases and antimicrobial stewardship independently reviewed and validated the assignment of codes, referencing previously published studies for accuracy and consistency.^8,11,12^ Some prescribers could specify ICD-10 when writing an antibiotic prescription, depending on the facility. Otherwise, one or more ICD-10 codes were listed during a given antibiotic-prescribing encounter, including the diagnosis resulting in an antibiotic prescription and other diagnoses observed during the visit. In those cases, we used a three-tier approach to identify a diagnosis most likely responsible for a given antibiotic prescription.^13,14^ This approach involves assigning one of the following categories to each diagnosis: (1) Tier 1: antibiotic definitely needed, (2) Tier 2: antibiotic sometimes needed or empirically used in practice, or (3) Tier 3: antibiotic would not be used. If a prescription was associated with multiple diagnoses with different tiers, lower-tier diagnoses were assumed to be responsible for the prescription, and higher-tier diagnoses were disregarded. If a prescription was associated with multiple tier 1 diagnoses, we examined whether the prescribed antibiotic was recommended by published guidelines for each diagnosis. If so, the diagnosis was assumed to be responsible for the prescription. If a prescribed antibiotic was recommended for two or more diagnoses, we assumed the antibiotic would treat all applicable diagnoses concurrently.

To assess the concordance of antibiotic prescriptions with recommendations from published guidelines,^15–23^ we focused on selected diagnoses for which antibiotics are commonly prescribed in outpatient settings. Those were urinary tract infections (UTIs), sinusitis, cellulitis, Group A Streptococci (GAS), and otitis media. The UTI group did not include pyelonephritis to account for differences in clinical management and recommended treatment durations. The list of ICD-10 codes for these conditions is shown in Table S1. Each antibiotic prescription was compared to the recommended regimen with respect to (1) antibiotic and (2) duration.

The quarterly rate of antibiotic prescriptions per 1 000 prescription-related outpatient visits and the proportion of prescriptions given to adults (age 18 or older) and children (younger than 18) were estimated. The top 10 most frequently prescribed antibiotics were identified for adults and children separately. The quarterly rate of antibiotic prescribing per 1 000 prescription-related outpatient visits for selected diagnoses listed above was estimated. The top 5 most frequently prescribed antibiotics for the selected diagnoses were identified, and the mean and median duration of antibiotic therapy were estimated. The guideline concordance rate for each indication was calculated. Prescribing rates were assessed using a binomial 95% confidence interval (95% CI). The top 10 list of antibiotics with missing diagnosis data were identified. Two sets of sensitivity analyses were conducted. First, encounters with multiple diagnoses from the same tier were excluded when calculating the guideline concordance rates. Second, antibiotic prescriptions that were longer than 21 days were excluded from the analysis because they may be related to chronic antibiotic suppression.

Ferris State University Institutional Review Board deemed this study not human subjects research and, therefore, exempt from institutional review board review and approval. This study followed the Strengthening the Reporting of Observational Studies in Epidemiology (STROBE) reporting guideline.

## Result

**Figure 1.**
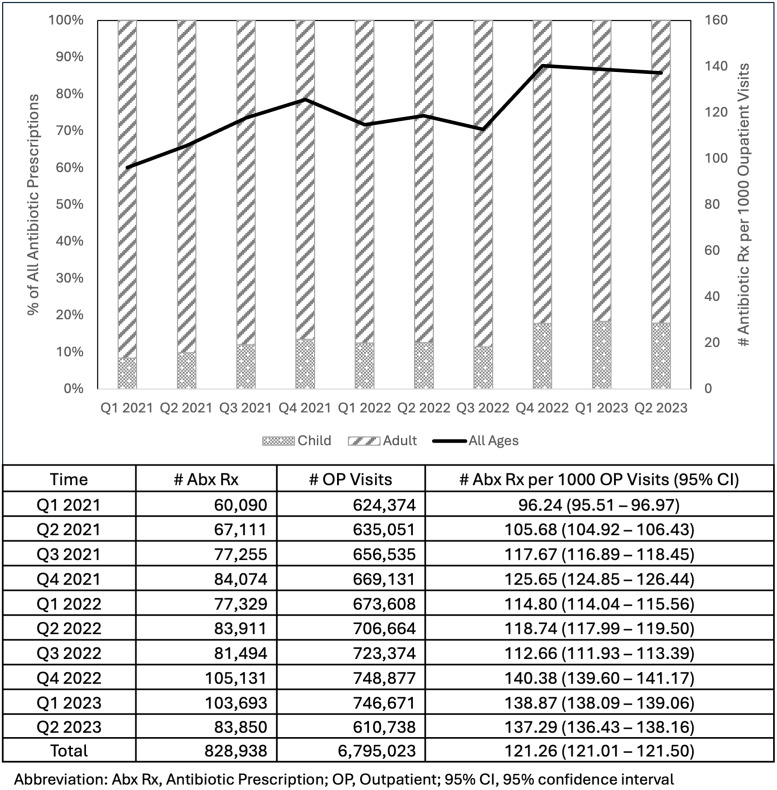
The rate of antibiotic prescribing in outpatient settings.

Out of 6,795,023 prescription-related outpatient visits between January 2021 and June 2023, 823,938 antibiotic prescriptions were prescribed, resulting in a rate of 121.26 per 1 000 prescription-related outpatient visits (95% CI 121.01–121.50). The quarterly rate of antibiotic prescriptions is shown in Figure 1. The rate increased from 96.24 per 1 000 in the first quarter of 2021 to 114.80 in the same quarter of 2022 (19% increase) and further increased to 138.87 in Q1 2023 (20% increase from 2022). On average, 13.4% (3.5%) of all antibiotic prescriptions were for children.

The top 10 most frequently prescribed antibiotics are listed in Table 1. These drugs accounted for 77% of all antibiotic prescriptions. Amoxicillin-clavulanate (12%), amoxicillin (12%), doxycycline (11%), and cephalexin (11%) were the most frequently prescribed antibiotics. There were variations between facilities for the top antibiotic; however, these drugs generally made up the top antibiotics in most facilities (facility-level data not shown). Doxycycline was more common in adults (13% of all adult prescriptions) than in children (2% of all child prescriptions). Ciprofloxacin was ranked 8^th^, accounting for 4% of all antibiotic prescriptions. Nearly 98% of all ciprofloxacin prescriptions were written for adults, and 51% were written for those aged 60 or older. Amoxicillin was most common in children (42% of all child prescriptions). Cefdinir was more common in children than adults, ranking 3^rd^ in children. Amoxicillin-clavulanate, cephalexin, and azithromycin appeared widely used in adults and children.

**Table 1.** Frequently prescribed antibiotics in outpatient settings

Rank	All Ages			Adult (Age 18 or Older)			Child (Younger than Age 18)		
	Antibiotic	# Prescriptions	% of All	Antibiotic	# Prescriptions	% of All	Antibiotic	# Prescriptions	% of All
1	AMX-CLV	97,805	12%	Doxycycline	89,556	13%	Amoxicillin	47,653	42%
2	Amoxicillin	95,657	12%	AMX-CLV	86,242	12%	AMX-CLV	11,563	10%
3	Doxycycline	92,176	11%	Cephalexin	76,927	11%	Cefdinir	10,390	9%
4	Cephalexin	86,611	11%	Azithromycin	60,434	9%	Cephalexin	9,684	8%
5	Azithromycin	69,870	8%	SMZ-TMP	50,008	7%	Azithromycin	9,436	8%
6	SMZ-TMP	53,637	7%	Amoxicillin	48,004	7%	SMZ-TMP	3,629	3%
7	Nitrofurantoin	45,209	5%	Nitrofurantoin	44,394	6%	Doxycycline	2,620	2%
8	Ciprofloxacin	29,898	4%	Ciprofloxacin	29,543	4%	Clindamycin	1,559	1%
9	Metronidazole	28,787	3%	Metronidazole	28,281	4%	Minocycline	907	1%
10	Cefdinir	21,244	3%	Clindamycin	15,876	2%	Nitrofurantoin	815	1%
	All Antibiotics	823,938		All Antibiotics	709,348		All Antibiotics	114,590	

Abbreviation: AMX-CLV, amoxicillin-clavulanate; SMZ-TMP, sulfamethoxazole-trimethoprim.

There were 660,516 prescriptions with diagnosis information (80% of all antibiotic prescriptions). Nearly half (*n* = 328,876) were directly linked to a specific diagnosis by the prescriber when they wrote the prescription. For prescriptions with multiple diagnoses not explicitly linked to the antibiotic (*n* = 331,640), we used the three-tier approach described in Methods. As a result, 109,414 prescriptions for sinusitis, 77,242 prescriptions for UTIs, 60,019 prescriptions for otitis media, 42,446 prescriptions for GAS, and 35,938 prescriptions for cellulitis were identified, adults and children combined. The list of antibiotics with missing diagnoses is shown in Table S2. The top 9 antibiotics in Table 1 are consistently present in the top 9 of Table S2, although their ranks differ. The most frequently prescribed antibiotics remained the same, regardless of the presence or absence of diagnosis data.

The quarterly antibiotic prescribing rates for the diagnoses among adults were analyzed. (Figure S1). The rate for sinusitis and UTIs were consistently high during the observed period. The rate for sinusitis was highest in the last quarter of each year (13.19 per 1 000 prescription-related outpatient visits in Q4 2021 and 14.88 in Q4 2022), with an upward trend. The rate for UTIs peaked at 12.99 in Q3 2021 and remained relatively high, with a slight decrease to 10.62 in Q1 2023. The rate for cellulitis showed variability but generally remained between 4.06 and 5.81 in 2022–2023. The rate for GAS increased from 1.58 in Q1 2021 to 7.34 in Q1 2023. In children, the rate of antibiotic prescriptions for otitis media showed a marked increase from 1.44 in Q1 2021 to 10.89 in Q4 2022, followed by a decrease to 6.34 in Q2 2023 (Figure S2). Between the third and fourth quarters of 2022, the rate increased by 152%, from 4.33 to 10.89. A notable increase was also observed in GAS-related prescribing, from 2.94 in Q4 2022 to 7.80 in Q1 2023 (165% increase). Prescribing for cellulitis and UTI remained low (<1.00 per 1 000) throughout the period.

The top five drugs and their durations for the selected diagnoses for adults and children are shown in Tables 2 and 3, respectively. The top five drugs accounted for a majority of prescriptions in each diagnosis. Among adults, sinusitis was the most common reason for prescribing antibiotics. Amoxicillin-clavulanate comprised 31% of all sinusitis-related prescriptions, followed by doxycycline (15%). Both drugs were considered guideline-concordant for sinusitis.^19^ Azithromycin, a discordant antibiotic for sinusitis, was prescribed 9% of the time.

**Table 2.** Top five drugs and their durations for selected indications in adults

Indication Group	Rank	Drug	*n*	% of Indication Total	Drug Concordance	Duration Days, Mean (Median)	Recommended Duration Days	Reference
Sinusitis (n = 100,265)	1	AMX-CLV	30,585	31%	Concordant	8.78 (7)	5–7	^19^
	2	Doxycycline	14,609	15%	Concordant	8.69 (7)	5–7	^19^
	3	Azithromycin	9,271	9%	Discordant	9.78 (5)		
	4	Amoxicillin	7,193	7%	Concordant	9.09 (7)	5–10	^20^
	5	Cefdinir	2,064	2%	Concordant	9.08 (10)	5–7	^23^
Urinary Tract Infection (*n* = 73,095)	1	Nitrofurantoin	19,032	26%	Concordant	6.81 (5)	5–7	^17^
	2	Cephalexin	18,565	25%	Concordant	5.96 (7)	5–7	^23^
	3	SMZ-TMP	11,261	15%	Concordant	6.18 (7)	3	^17^
	4	Ciprofloxacin	8,671	12%	Concordant	7.48 (7)	3	^17,18^
	5	Metronidazole	4,063	6%	Discordant	7.88 (7)		
Cellulitis (*n* = 32,077)	1	Cephalexin	11,507	36%	Concordant	7.86 (7)	5	^15^
	2	Doxycycline	6,437	20%	Concordant	8.28 (7)	5	^15^
	3	AMX-CLV	4,299	13%	Discordant	9.25 (7)		
	4	SMZ-TMP	4,071	13%	Concordant	8.63 (7)	5	^15^
	5	Clindamycin	1,697	5%	Concordant	12.57 (7)	5	^15^
Group A Streptococci (*n* = 24,725)	1	Amoxicillin	10,248	42%	Concordant	9.00 (7)	10	^16^
	2	Azithromycin	4,114	17%	Concordant	7.79 (5)	5	^16^
	3	AMX-CLV	3,991	16%	Discordant	9.21 (7)		
	4	Cephalexin	1,160	5%	Concordant	9.21 (7)	10	^16^
	5	Penicillin V	2,100	9%	Concordant	9.12 (10)	10	^16^
Otitis Media (*n* = 22,205)	1	AMX-CLV	7,766	35%	Concordant	9.11 (7)	7–10	^21^
	2	Amoxicillin	5,617	25%	Concordant	7.98 (7)	5–10	^21^
	3	Azithromycin	2,224	10%	Discordant	9.11 (5)		
	4	Cefdinir	1,751	8%	Concordant	8.72 (7)	5–7	^21^
	5	Doxycycline	1,315	6%	Discordant	8.42 (7)		

Abbreviation: AMX-CLV, amoxicillin-clavulanate; SMZ-TMP, sulfamethoxazole-trimethoprim.

**Table 3.** Top five drugs and their durations for selected indications in children

Indication Group	Rank	Antibiotic	*n*	% of Indication Total	Drug Concordance	Duration Days, Mean (Median)	Recommended Duration Days	Reference
Otitis Media (*n* = 37,814)	1	Amoxicillin	23,923	63%	Concordant	9.04 (7)	5–10	^22^
	2	Cefdinir	6,071	16%	Concordant	9.40 (7)	5–10	^22^
	3	AMX-CLV	4,495	12%	Concordant	9.27 (7)	5–10	^22^
	4	Azithromycin	2,566	7%	Discordant	8.97 (5)		
	5	Cephalexin	175	.5%	Discordant	7.44 (7)		
Group A Streptococci (*n* = 17,721)	1	Amoxicillin	11,945	67%	Concordant	9.17 (7)	10	^16^
	2	Azithromycin	1,813	10%	Concordant	6.33 (5)	5	^16^
	3	Cephalexin	1,064	6%	Concordant	8.76 (7)	10	^16^
	4	AMX-CLV	1,034	6%	Discordant	8.76 (7)		
	5	Cefdinir	870	5%	Discordant	8.32 (7)		
Sinusitis (*n* = 9,149)	1	Amoxicillin	2,531	28%	Concordant	9.77 (7)	5–10	^20^
	2	AMX-CLV	2,060	23%	Concordant	9.06 (7)	10–14	^19^
	3	Azithromycin	879	10%	Discordant	4.28 (5)		
	4	Cefdinir	736	8%	Concordant	10.59 (10)	10–14	^19^
	5	Doxycycline	237	3%	Discordant	8.00 (10)		
Urinary Tract Infection (*n* = 4,147)	1	Cephalexin	1,632	39%	Concordant	6.84 (7)	5–10	^23^
	2	SMZ-TMP	973	23%	Concordant	7.19 (7)	3	^17^
	3	Cefdinir	496	12%	Concordant	6.07 (7)	3–7	^17^
	4	Nitrofurantoin	436	11%	Concordant	5.95 (5)	5–7	^17^
	5	Amoxicillin	197	5%	Discordant	6.87 (7)		
Cellulitis (*n* = 3,861)	1	Cephalexin	1,694	44%	Concordant	7.21 (7)	5	^15^
	2	AMX-CLV	761	20%	Discordant	7.40 (7)		
	3	SMZ-TMP	555	14%	Discordant	7.69 (7)		
	4	Clindamycin	142	4%	Concordant	9.86 (7)	5	^15^
	5	Doxycycline	140	4%	Discordant	6.73 (7)	5	^15^

Abbreviation: AMX-CLV, amoxicillin-clavulanate; SMZ-TMP, sulfamethoxazole-trimethoprim.

Nitrofurantoin (26%) and cephalexin (25%) accounted for nearly half of prescriptions for UTIs. Sulfamethoxazole-trimethoprim (15%) and ciprofloxacin (12%) were also frequently used for UTIs. Based on the average and median, these drugs tended to be prescribed for longer than the recommended 3-day duration.^17,18^

For cellulitis, cephalexin and doxycycline accounted for 56% of all prescriptions. While both drugs were recommended per guidelines,^15^ the average durations were longer than the recommended five days. Amoxicillin was primarily prescribed for GAS (42%) and amoxicillin-clavulanate for otitis media (35%).

Overall, 56% of sinusitis-related prescriptions in adults included a concordant antibiotic (Table 4). When duration is taken into account, 30% of all prescriptions were concordant. Of UTI-related prescriptions, 87% were antibiotic concordant, and 52% were antibiotic and duration concordant. 79% of cellulitis-related prescriptions were antibiotic concordant, but 7% were within the recommended 5-day duration. Approximately 84% of cellulitis-related prescriptions were for 7–14 days (data not shown).

**Table 4.** Drug and duration concordance for selection indications

Age Group	Indication Group	# Prescriptions with drug and duration data (A)	# Prescriptions with Concordant Drug (B)	# Prescriptions with Concordant Duration (C)	% Drug Concordant (B/A)	% Duration Concordant (C/B)	% Drug and Duration Concordant (C/A)
Adult	Sinusitis	99,990	55,871	30,311	56%	54%	30%
	Urinary Tract Infection	70,761	61,274	36,580	87%	60%	52%
	Cellulitis	31,049	24,410	2,270	79%	9%	7%
	Group A Streptococci	22,806	18,179	11,033	80%	61%	48%
	Otitis Media	21,573	15,585	12,946	72%	83%	60%
Child	Otitis Media	35,556	34,488	30,517	97%	88%	86%
	Group A Streptococci	16,010	15,470	9,097	97%	59%	57%
	Sinusitis	9,041	5,429	3,782	60%	70%	42%
	Urinary Tract Infection	4,060	3,660	2,221	90%	61%	55%
	Cellulitis	3,770	1,838	261	49%	14%	7%

The antibiotic prescribing trend for children is shown in Table 3. Upper respiratory tract infections, including otitis media, GAS, and sinusitis, were the primary reasons children received antibiotic prescriptions. Amoxicillin was the most widely used antibiotic for these diagnoses (63%, 67%, and 28% of prescriptions in each group, respectively), with a median duration of 7 days. Cefdinir, amoxicillin-clavulanate, and azithromycin were also used for these indications. Approximately 86% of otitis media-related prescriptions were antibiotic and duration concordant (Table 4). 97% of GAS-related prescriptions were antibiotic concordant, and 57% were antibiotic and duration concordant. Similar to adults, 55% of UTI-related prescriptions and 7% of cellulitis-related prescriptions were antibiotic and duration concordant in children.

In sensitivity analyses, excluding encounters with multiple diagnoses from the same tier did not alter the concordance rates for UTI, cellulitis, GAS, and otitis media by more than 5% compared to the primary analysis (Table S3). However, the drug concordance rates for sinusitis increased by over 25% in both adults and children. Additionally, when prescriptions with durations longer than 21 days were excluded, the sinusitis drug concordance rates improved by more than 30% in both adults and children (Table S4). For other diagnoses, the differences were less than 5%.

## Discussion

Antimicrobial stewardship plays a critical role in healthcare, aiming to improve antibiotic use and prevent resistance. Analyzing prescribing patterns is the initial step toward developing effective interventions. This study analyzed over 800,000 antibiotic prescriptions from more than 300 outpatient facilities between Q1 2021 and Q2 2023 to assess the trend and guideline concordance. Antibiotic use in the outpatient setting fluctuated, gradually increasing when comparing the same quarter each year. Upper respiratory tract infections such as sinusitis and otitis media were the most common reasons for prescribing antibiotics in outpatient settings. The COVID-19 pandemic’s influence on outpatient antibiotic prescribing patterns was likely substantial. In our study, the antibiotic prescribing rate was notably low in 2021, likely due to pandemic-related factors such as reduced in-person visits, increased telehealth services, and widespread public health measures such as social distancing and mask-wearing. These measures led to a decline in respiratory infections and other communicable diseases, reducing the need for antibiotics. However, as the pandemic progressed and healthcare systems adapted, the antibiotic use rate gradually increased, reaching higher levels by Q2 2023. This overall increase may reflect a return to prepandemic healthcare utilization patterns and a resurgence in infections as public health measures were relaxed.

One of the key strengths of this study is that it analyzes EHR data extracted from various outpatient facilities. Each facility’s EHR system may have unique data structures and coding practices, making the harmonization of data a complex and meticulous process. Our study integrated diverse data sources to analyze antibiotic prescribing trends across multiple facilities. This approach enhances the robustness of our findings and the reliability of the observed trends. For example, a pattern of antibiotic use similar to our observations was reported by Bizune et al., utilizing the IQVIA National Prescription Audit (NPA) Extended Insights data set, which encompasses nationwide antibiotic prescription transactions in retail pharmacies across the United States.^24^ The overall increase in antibiotic use and the quarterly trends from 2021 to 2022 are comparable. As in their study, we observed a gradual increase in antibiotic use from the first quarter to the last quarter of 2021. From the last quarter of 2021 to the first quarter of 2022, there was a reduction, followed by a stable rate until the fourth quarter, which saw the sharpest increase since the first quarter of 2021. These consistent trends across different datasets highlight the reliability of our findings and the broader applicability of our study’s conclusions.

Our analysis captures important trends in antibiotic prescribing, emphasizing the need for continued surveillance and stewardship efforts. The increasing rate of antibiotic prescriptions, particularly among children, underscores the need for ongoing monitoring and intervention. The prescription rate for otitis media increased by 152% in the last quarter of 2022, and GAS increased by 165% in the first quarter of 2023 compared to their respective previous quarters. This trend coincides with the 2022–2023 viral respiratory disease season, during which COVID-19, influenza, and respiratory syncytial virus (RSV) infection rates were elevated.^25^ The increased incidence of otitis media and GAS infections could have been complications of these viral infections.^26^ The elevated viral respiratory infections and the corresponding surge in antibiotic prescriptions raise questions about the appropriateness of these treatments. Viral infections are often self-limiting and do not require antibiotic therapy; however, healthcare providers might prescribe antibiotics preemptively against secondary bacterial infections.^27^ Also, if the diagnosis of bacterial and viral infections is uncertain, overprescribing could occur as a precautionary measure. This practice, although it is sometimes necessary, emphasizes the need for advanced diagnostic techniques to assist with clinical decision-making.

The discrepancies observed in antibiotic and duration concordance with guidelines indicate substantial room for improvement. Less than 60% of sinusitis-related prescriptions were antibiotic concordant. Nearly 10% of sinusitis-related prescriptions were azithromycin in adults and children, despite guidelines advising against its use for sinus infections.^19,20^ Longer than recommended duration contributed to low concordance rates in UTIs, GAS, and cellulitis. While sometimes prescriptions may have extended durations based on clinical improvement, we observed that prescriptions with an extended duration are prevalent. For example, over 80% of cellulitis-related prescriptions were for 7–14 days, compared to the recommended 5-day course.^15^

Ciprofloxacin’s frequent use for UTIs and the tendency to prescribe it to older adults indicate another critical gap in practice. Ciprofloxacin was the fourth most used drug for UTIs, accounting for 12% of all UTI-related prescriptions in adults. Notably, more than half of ciprofloxacin prescriptions were provided to individuals aged 60 or older. The overuse of fluoroquinolones, such as ciprofloxacin, is known to increase the risk of severe and potentially irreversible long-term adverse side effects, such as tendon rupture and peripheral neuropathy, especially in older adults.^28^ There is also a greater risk of developing antimicrobial resistance with these broad-spectrum antibiotics.^29^ Future studies should investigate factors affecting the prescribing of fluoroquinolone and develop appropriate interventions to reduce its use.

While improving antibiotic prescribing in outpatient settings is crucial for patient safety, several challenges exist. Many healthcare providers may lack awareness of antibiotic resistance issues surrounding outpatient care or have insufficient knowledge about appropriate antibiotic prescribing practices.^4,30,31^ Diagnostic uncertainties and limited abilities to conduct a test create challenges to optimal antibiotic treatment.^32^ Conducting a test to make a definitive diagnosis may not be feasible in some settings, especially if the providers have limited time to see patients.^32^ The fear of undertreatment and the motivation to satisfy patient expectations may add pressure to overprescribe antibiotics.^33,34^ Patients may expect or demand antibiotics when they visit providers, and it can be difficult for providers to manage these expectations under pressure to achieve better patient satisfaction. Studies have shown that reducing diagnostic uncertainty through point-of-care testing (POCT) could decrease unnecessary antibiotic use.^35^ Delayed prescribing, a practice in which a patient receiving an antibiotic prescription is instructed to fill it later if their symptoms do not improve, has been shown to reduce antibiotic use in outpatient settings.^36–38^

The study’s methodology, including the use of a three-tier approach to classifying diagnoses and the assessment of prescribing concordance with guidelines, provides a robust framework for assessing prescribing practices and opportunities to benchmark. However, the reliance on EHR data means that the accuracy of the diagnosis and prescribing information depends on the quality of the data entered.^39^ Unique clinical cases may warrant antibiotic treatments that deviate from guidelines. Some instances of guideline discordance, such as the use of metronidazole for UTIs, may reflect clinically appropriate decisions that were not discernible in our data. Furthermore, this study did not capture delayed antibiotic prescribing or whether prescriptions were filled. Other factors, such as dosage adjustment for renal function, consideration of drug allergies, and the appropriate testing procedure, were not assessed.

The sensitivity analyses provided valuable insights into the implications of analytical approaches, particularly for sinusitis. The substantial increase in drug concordance rates for sinusitis when encounters with multiple diagnoses from the same tier were excluded suggests that diagnostic coding complexities may have influenced our primary results. Additionally, the notable improvement in drug concordance for sinusitis after excluding prescriptions with durations exceeding 21 days indicates that unusually long durations may have contributed to the lower concordance observed in the primary analysis. These findings highlight the importance of refining analytic approaches to account for potential misclassification and outlier prescription patterns in sinusitis treatment. On the other hand, the minimal changes observed for UTI, cellulitis, GAS, and otitis media suggest that the initial estimates for these conditions were less sensitive to alternative analytic decisions.

To enhance the interpretability of the results, we focused on prescription-related outpatient visits to estimate the antibiotic prescribing rates. Including all visits, regardless of whether a prescription was issued, could introduce noise into the data, as fluctuations in visits that do not require treatment, such as wellness visits and follow-ups, could obscure actual prescribing trends. However, it should be noted that care-seeking patterns may indirectly affect our findings. During the COVID-19 pandemic, the number of outpatient visits with upper respiratory infections not requiring antibiotic prescriptions increased.^14^ If providers altered their prescribing thresholds in response to changes in patient volume or illness severity, this could influence observed trends. Our estimates reflect prescribing among visits that resulted in a prescribing and may not capture broader changes in care-seeking behavior or treatment thresholds. Additionally, our data does not account for over-the-counter medication use or treatment delays, which could affect the overall antibiotic prescribing landscape.

Clinical guidelines are not always updated frequently, and some recommendations may not fully reflect the most recent evidence. While we strictly adhered to published guidelines from authoritative bodies, some of these guidelines were developed several years ago and have not been revised to incorporate more recent findings on optimal antibiotic duration. For this reason, there may be instances where a prescription classified as concordant in our analysis would be considered excessive by more current best practices. The aggregate data reported in this study should not be extrapolated to make inferences about local practices. A number of local factors can influence antibiotic selection, including access to POCT or other diagnostic techniques, the prescriber’s training and specialty, local antimicrobial resistance patterns, and the characteristics of the local patient population. ASPs must review their facility-specific data and tailor interventions to address the facility-specific practices and challenges.^40^ A more tailored approach is needed to ensure ASP initiatives are relevant and responsive.^40^

The findings of this study highlight the urgency of bolstering ASP efforts in outpatient settings. By analyzing antibiotic use trends and identifying areas for intervention, healthcare systems can implement more effective stewardship strategies. The increasing rate of antibiotic prescriptions, particularly among children, and the discrepancies in guidelines concordance emphasize the need for ongoing monitoring and interventions. Future research should focus on understanding the factors influencing prescribing behaviors and developing targeted interventions to promote appropriate antibiotic use. Strengthening ASPs and ensuring data-informed decision-making are critical steps toward mitigating antibiotic resistance and improving patient care.

## Supporting information

10.1017/ash.2025.10081.sm001 Sohn et al. supplementary material Sohn et al. supplementary material

## Supplementary material

The supplementary material for this article can be found at https://doi.org/10.1017/ash.2025.10081.

## Research Transparency and Reproducibility

Due to agreements with data providers, the raw data underlying this research are not publicly available.
