# Supplementary material for: Outpatient antibiotic stewardship during the COVID-19 era: analysis of prescribing trends and guideline compliance
Source: Antimicrob Steward Healthc Epidemiol. 2025 Aug 4;5(1):e168. doi: 10.1017/ash.2025.10081 (PMC12322779; doi:10.1017/ash.2025.10081)
Supplement: Sohn et al. supplementary material [file S2732494X25100818sup001.docx]

**Supplementary Materials**

**Outpatient Antibiotic Stewardship: Analysis of Prescribing Trends and Compliance with Guidelines**

Table S1. Identification of diagnosis groups

| Diagnosis Group | ICD-10 |
| --- | --- |
| CELLULITIS | A28.0, A46, A49, E08.62, E10.62, E11.62, E13.621, H60.1, L03, L88, L89, L97, L98.4, P38, S01, S31, S41, S51, S61, S71, S81, S91, T87.89, W50.3, W53, W54, W55, W56, W57, W58, W59, W60, W61, Y04 |
| GROUP A STREPTOCOCCI | A38, A49.1, B95.0, I00, I05, I07, I08, I09, J02, J03, J04, J35, J36, J39.0, J39.1, J39.2, R07.0 |
| OTITIS MEDIA | H65, H66, H67, H68.0, H68.1, H69.8, H69.9, H72.9, H73.00, H73.01, H73.1, H73.2, H73.89, H73.9, H74.0, H92, H93.8X, H93.9 |
| SINUSITIS | J01, J32, R09.81, R09.82 |
| URINARY TRACT INFECTION | A54.9, B96.1, B96.2, B96.4, N02, N30.0, N30.2, N30.3, N30.8, N30.9, N31.2, N31.8, N31.9, N32.0, N34.0, N34.1, N34.2, N39.0, N39.41, N39.42, N70.9, N71, N72, N73, N75, N76, N90.7, O23.1, O23.4, O23.59, O26.89, O86.12, O86.19, O86.20, O86.22, O86.29, O99.89, R30, R31, R33.8, R33.9, R35, R39.15, R82.7, R82.8, R82.9, T83.51, T83.59 |

Figure S1. The quarterly rate of antibiotic prescribing for selected indications in adults

| Time | # Antibiotic Prescriptions per 1000 Outpatient Visits | | | | |
| --- | --- | --- | --- | --- | --- |
|  | CELLULITIS | GROUP A STREPTOCOCCI | OTITIS MEDIA | SINUSITIS | URINARY TRACT INFECTION |
| Q1 2021 | 3.04 | 1.58 | 1.82 | 5.47 | 7.86 |
| Q2 2021 | 5.17 | 2.04 | 2.02 | 6.99 | 10.81 |
| Q3 2021 | 6.35 | 2.97 | 3.06 | 8.06 | 12.99 |
| Q4 2021 | 4.56 | 3.61 | 3.89 | 13.19 | 11.85 |
| Q1 2022 | 4.06 | 2.83 | 3.50 | 12.23 | 10.25 |
| Q2 2022 | 5.16 | 3.10 | 3.35 | 10.27 | 10.77 |
| Q3 2022 | 5.81 | 2.50 | 2.97 | 6.43 | 11.90 |
| Q4 2022 | 4.21 | 4.22 | 4.58 | 14.88 | 10.77 |
| Q1 2023 | 4.22 | 7.34 | 4.09 | 13.92 | 10.62 |
| Q2 2023 | 5.50 | 6.41 | 3.51 | 9.30 | 11.43 |

Figure S2. The quarterly rate of antibiotic prescribing for selected indications in children

| Time | # Antibiotic Prescriptions per 1000 Outpatient Visits | | | | |
| --- | --- | --- | --- | --- | --- |
|  | CELLULITIS | GROUP A STREPTOCOCCI | OTITIS MEDIA | SINUSITIS | URINARY TRACT INFECTION |
| Q1 2021 | 0.31 | 0.62 | 1.44 | 0.36 | 0.44 |
| Q2 2021 | 0.54 | 0.68 | 2.65 | 0.52 | 0.63 |
| Q3 2021 | 0.80 | 1.62 | 4.46 | 0.67 | 0.71 |
| Q4 2021 | 0.47 | 1.73 | 6.72 | 1.48 | 0.67 |
| Q1 2022 | 0.38 | 1.31 | 5.91 | 1.11 | 0.62 |
| Q2 2022 | 0.63 | 1.28 | 5.90 | 0.95 | 0.54 |
| Q3 2022 | 0.74 | 1.09 | 4.33 | 0.54 | 0.61 |
| Q4 2022 | 0.53 | 2.94 | 10.89 | 1.87 | 0.62 |
| Q1 2023 | 0.60 | 7.80 | 8.33 | 1.34 | 0.70 |
| Q2 2023 | 0.71 | 6.78 | 6.34 | 0.79 | 0.70 |

Table S2. Top 10 Antibiotics with Missing Diagnoses

| Rank | Antibiotic | # Prescriptions | % of All |
| --- | --- | --- | --- |
| 1 | Doxycycline | 16,377 | 10% |
| 2 | Amoxicillin | 13,196 | 8% |
| 3 | Nitrofurantoin | 12,625 | 8% |
| 4 | Cephalexin | 12,463 | 8% |
| 5 | Sulfamethoxazole/Trimethoprim | 11,462 | 7% |
| 6 | Azithromycin | 10,949 | 7% |
| 7 | Amoxicillin/Clavulanate | 10,372 | 6% |
| 8 | Metronidazole | 8,089 | 5% |
| 9 | Ciprofloxacin | 7,011 | 4% |
| 10 | Clindamycin | 3,499 | 2% |
|  | All Prescriptions Missing Diagnosis | 163,422 |  |

Table S3. Drug and Duration Concordance for Selection Indications – Excluding Encounters with Multiple Diagnoses from the Same Tier

| Age Group | Indication Group | # Prescriptions with drug and duration data (A) | # Prescriptions with Concordant Drug (B) | # Prescriptions with Concordant Duration (C) | % Drug Concordant (B/A) | % Duration Concordant (C/B) | % Drug and Duration Concordant (C/A) |
| --- | --- | --- | --- | --- | --- | --- | --- |
| Adult | Sinusitis | 64,629 | 53,445 | 29,206 | 83% | 55% | 45% |
|  | Urinary Tract Infection | 59,996 | 54,245 | 33,629 | 90% | 62% | 56% |
|  | Cellulitis | 24,251 | 19,504 | 1,899 | 80% | 10% | 8% |
|  | Group A Streptococci | 21,170 | 17,070 | 10,646 | 81% | 62% | 50% |
|  | Otitis Media | 18,574 | 14,112 | 12,009 | 76% | 85% | 64.7% |
| Child | Otitis Media | 33,057 | 31,410 | 28,654 | 95% | 91% | 87% |
|  | Group A Streptococci | 14,469 | 14,110 | 8,614 | 98% | 61% | 60% |
|  | Sinusitis | 5,619 | 4,835 | 3,240 | 86% | 67% | 58% |
|  | Urinary Tract Infection | 3,534 | 3,272 | 2,042 | 93% | 62% | 58% |
|  | Cellulitis | 2,979 | 1,551 | 220 | 52% | 14% | 7% |

Table S4. Drug and Duration Concordance for Selection Indications – Excluding Prescriptions with Durations Exceeding 21 Days

| Age Group | Indication Group | # Prescriptions with drug and duration data (A) | # Prescriptions with Concordant Drug (B) | # Prescriptions with Concordant Duration (C) | % Drug Concordant (B/A) | % Duration Concordant (C/B) | % Drug and Duration Concordant (C/A) |
| --- | --- | --- | --- | --- | --- | --- | --- |
| Adult | Sinusitis | 61,561 | 53,795 | 30,311 | 87% | 56% | 49% |
|  | Urinary Tract Infection | 63,155 | 56,555 | 36,580 | 90% | 65% | 58% |
|  | Cellulitis | 28,742 | 23,021 | 2,270 | 80% | 10% | 8% |
|  | Group A Streptococci | 20,201 | 16,113 | 11,033 | 80% | 68% | 55% |
|  | Otitis Media | 19,554 | 14,925 | 12,946 | 76% | 87% | 66% |
| Child | Otitis Media | 33,165 | 31,806 | 30,517 | 96% | 96% | 92% |
|  | Group A Streptococci | 13,925 | 13,562 | 9,097 | 97% | 67% | 65% |
|  | Sinusitis | 5,461 | 5,067 | 3,789 | 93% | 75% | 69% |
|  | Urinary Tract Infection | 3,811 | 3,468 | 2,218 | 91% | 64% | 58% |
|  | Cellulitis | 3,540 | 1,736 | 261 | 49% | 15% | 7% |
